# Supplementary material for: Cell-autonomous control coupled with tissue context regulates the cessation of migration at the site of organ development
Source: Development. 2026 Feb 20;153(16):dev205271. doi: 10.1242/dev.205271 (PMC12967139; doi:10.1242/dev.205271)
Supplement: Supplementary information [file develop-153-205271-s1.pdf]

Supplementary Materials and Methods

RNA tomography (Tomo-seq)

RNA from each section was extracted using 0.5 ml TRIzol reagent (Thermo Fisher) (at RT) mixed with 0.5 µl GlycoBlue (Ambion) and 1 µl of ERCC of spike-in (Ambion) diluted 1:50,000. After 15 seconds of vigorous shaking and 5 minutes of incubation at RT, 100 µl of chloroform was added to each sample and followed by 15 seconds of vigorous shaking and 5 minutes of incubation at RT and centrifugation at 12,000 g for 15 minutes at 4°C. The aqueous supernatant was transferred to LoBind 1.5 ml tubes (Eppendorf) with 250 µl isopropanol and were vigorously shaken for 15 seconds and incubated at -20°C for 1 h to ON. Next, the samples were spun down at 12,000 g for 10 minutes at 4°C. The supernatant was removed, and the pellet was washed with 75% ethanol (7,500 g for 5 minutes at 4 °C). At last, the pellet was air dried at RT. This step was followed by reverse transcription and *in vitro* transcription with the MessageAmpII kit (Ambion) following the CEL-seq method with minor modifications as described previously (Hashimshony et al., 2012) and following the instructions of the MessageAmpII kit. The first step was first strand synthesis using barcoded primers (5 ng/µl). To each sample one barcoded primer was added. This was followed by the second strand synthesis and cDNA clean-up. Prior to cDNA clean-up all the samples were pooled together. Next, cDNA amplification was performed by *in vitro* transcription of the cDNA. Afterwards, 5.5 µl RNA fragmentation buffer per sample was added, and the samples were incubated at 94°C for 3-6 minutes. The reaction was stopped by quickly transferring the samples on ice and addition of 2.75 µl of 0.5 M EDTA. The samples were then cleaned up using 1.8x Agencourt RNAClean XP beads. The concentration of the amplified RNA was measured using the Qubit RNA high sensitivity kit (Thermo Fisher) and RNA size distribution was assessed by an RNA High Sensitivity ScreenTape assay using a TapeStation system (Agilent). The RNA was diluted to a maximum of 50 ng/µL in a volume of 5 µL for the second reverse transcription (RT). 0.5 µL of the second RT primer (random hexamers, 250 ng/µL) and 0.5 µL dNTPs (10 mM total) were added to the RNA and incubated for 5 min at 65 °C, then immediately cooled on ice. Subsequently, first strand buffer (5X, Thermo Fisher), 1 µL DTT (0,1 M), 0.5 µL RNase Out and 0.5 µL SuperScript II reverse transcriptase

Development • Supplementary information

(Thermo Fisher) were added and the cDNA was generated. In a final PCR, the second Illumina sequencing adaptor and the respective indices were introduced. The resulting cDNA libraries was purified twice using Agencourt AMPure XP beads (Beckman Coulter). The DNA concentration and size distribution of the purified libraries were measured using the Qubit DNA high sensitivity assay kit and a D1000 ScreenTape® on an Agilent TapeStation system. The fragment distribution peaked at 200-500 bp. Two libraries for each sectioning direction were sequenced paired-end on an Illumina NextSeq500 using 75% of a High-Output flow cell. Read lengths were set to 70 for the first read (R1), containing the section barcoded and unique molecular identifier and 80 for the second read (R2) that is mapped to the transcriptome. Reads were aligned with STAR (Dobin et al., 2013) using STARsolo in droplet mode. Read counts of each gene were normalized to total counts per section. To find genes with a similar expression pattern to PGCs, *vasa (ddx4)* (PGC specific gene) was used as a reference gene and all the other genes were ranked based on their similarity to *vasa* expression pattern in 2D. The ranking was performed by calculating the Euclidean distance of Z-score transformed read counts of each gene in each section to *vasa.* Since tomo-seq was performed in two orientations (along the AP and DV axis), these data were combined by averaging the Euclidean distances and the read counts.

RNA sequencing (RNA-seq)

PGCs were sorted by fluorescence activated cell sorting (FACS) technique using a FACSAria IIIu cell sorter (BD Bioscience) equipped with a 70 µm nozzle at 15, 25 and 35 hpf into the lysis buffer. *Tg(kop:mCherry.F')* and *ody;Tg(kop:mCherry.F')* fishlines were used. Embryos at 15 and 25 hpf were collected and dechorionated by using 5 mg/ml protease (Sigma) while shaking for 6-8 minutes at RT. Next 100 embryos were collected in 2 ml tubes (Eppendorf) and incubated with 1 ml trypsin for 5-8 minutes at 31 °C. Trypsin was removed and the embryos were washed 2x with 500 µl (5 µl per embryo) of cell dissociation buffer (enzyme free, PBS based, Gibco). Afterwards, the embryos were dissociated in 400-500 µl of the cell dissociation buffer (Gibco) by gently pipetting up and down with a glass pipette (50-60 times). Next, the cell dissociation mixture was filtered through a 50 µm filter (Filtrix, 50 µm, Cytecs) and kept on ice until sorting. At 35 hpf, following dechorionation using 5 mg/ml protease (Sigma) (analogous to 15 and 25 hpf embryos) 100-120 embryos were collected in 2 ml tube

Development • Supplementary information

(Eppendorf) and were incubated with 1 ml of trypsin at 31 °C for 25 minutes. Next, embryos were disintegrated by slowly pipetting 40-60 times with a glass Pasteur pipette (Brand). The dissociated embryos were then filtered through a 50 µm filter (Filtrix, 50 µm, Cytecs) and were kept on ice. The flow through was then spun down at 400 g for 5 min (RT). Supernatant was removed and the pellet was resuspended in 400 - 500 µl of the cell dissociation buffer (Gibco) by pipetting for 10-20 times with a glass Pasteur pipette (Brand). This mixture was loaded on a 30 µm filter (Filtrix, 30 µm, Cytecs) into a 1.5 ml tube (Eppendorf) which was already cooled on ice. The filter was rinsed with 100 µl of the cell dissociation buffer (Gibco). The sample was kept on ice until cell sorting. The number of cells used for 15 hpf varied from 10000 - 13000, for 25 hpf from 8000 - 12000, and for 35 hpf from 6000 - 9000.

Total RNA was extracted using the PicoPure RNA extraction kit (Arcturus) following the manufacturer's instructions. The RNA was eluted in 30 µl of elution buffer (provided by the kit) and then purified using DNA-free kit (Thermo Fisher) 38 following the protocol provided by the manufacturer. 3 µl was used for checking the quality and concentration of the RNA and the rest was used for cDNA library preparation. The RNA was stored at -20 °C. mRNA was extracted from the total RNA using NEXTflex Poly(A) Beads (Hiss Diagnostics) following the manufacturer's instructions. 14 µl of the mRNA elute was transferred to a fresh 1.5 ml tube (Eppendorf). Next, cDNA was synthesized using the NEXTflex Rapid directional qRNA-Seq kit (Hiss Diagnostics) in six steps: RNA fragmentation, first strand synthesis, second strand synthesis, adenylation, adapter ligation, and PCR amplification. All the steps followed the manufacturer's protocol. After steps 3, 5 and 6 the solution was cleaned up using AMPure XP Magnetic Beads (Beckman Coulter) and following the manufacturer's protocol. In the last clean up step, the beads were rehydrated and resuspended in 14 µl of the resuspension buffer (included in the kit) followed by 2-minute incubation at RT and 5 min incubation on a magnetic stand until the supernatant was clear. 13 µl of the clear supernatant was transferred to a 1.5 ml tube (Eppendorf) and stored at -20 °C. 3 µl was used for determining the concentration and the quality of the cDNA library and the rest was used in the following steps. The Next-generation RNA-sequencing (RNA-seq) was performed on an Illumina NextSeq 550 machine using 2x 75 bp paired-end reads. To quantify transcripts from raw fastq files Salmon mapping-based mode (Patro et al., 2017) was used. Transcript information from Ensembl (Cunningham et al., 2019)

release 97 for Danio_rerio GRCz11 was utilized. Genes with less than ten reads in all samples were filtered out. Gene-level differential expression between groups was assessed by calculating the log2 of the fold change of the relevant stages. The threshold was set based on expression of PGC enriched genes such as *sod2* (Tarbashevich et al., 2023).

Development • Supplementary information

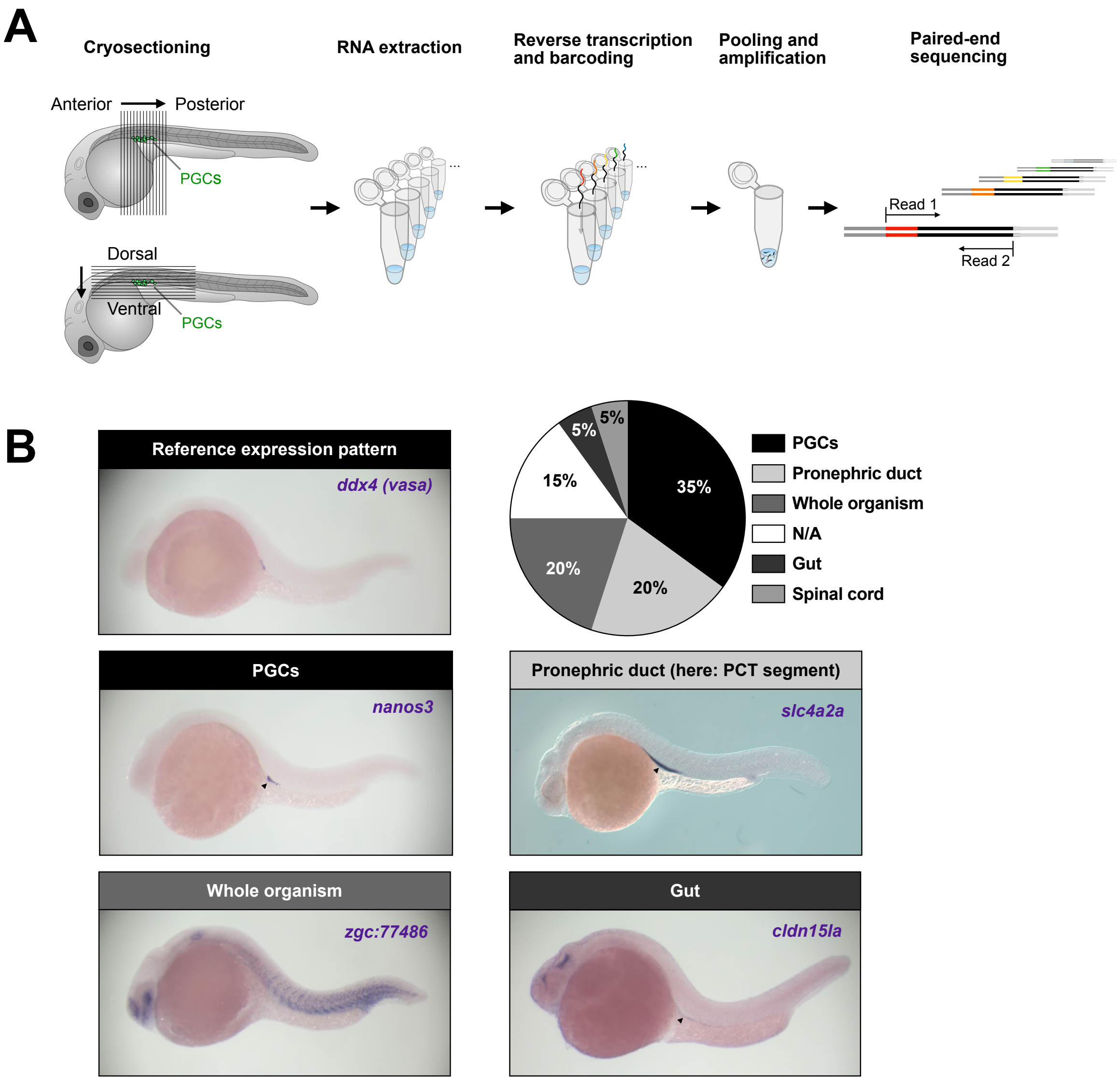

Fig. S1. RNA tomography identifies the pronephric duct as a structure adjacent to the PGCs. A. Schematic of the experimental setup. Embryos at 24 hpf were sectioned along the anterior–posterior and dorsal–ventral body axes to fully cover the gonadal ridge, where PGCs are located at this stage. RNA from individual sections was extracted, barcoded, and used for paired-end sequencing library preparation. **B.** Genes were ranked by the similarity of their expression profiles to the germ cell marker *ddx4* (formerly *vasa*) using Euclidean distance analysis. The 20 top ranking (most similar) genes were grouped according to the anatomical structures in which they are expressed, and the relative prevalence of these structures was compared. Representative whole-mount *in situ* hybridization images illustrate expression patterns in the respective tissues.

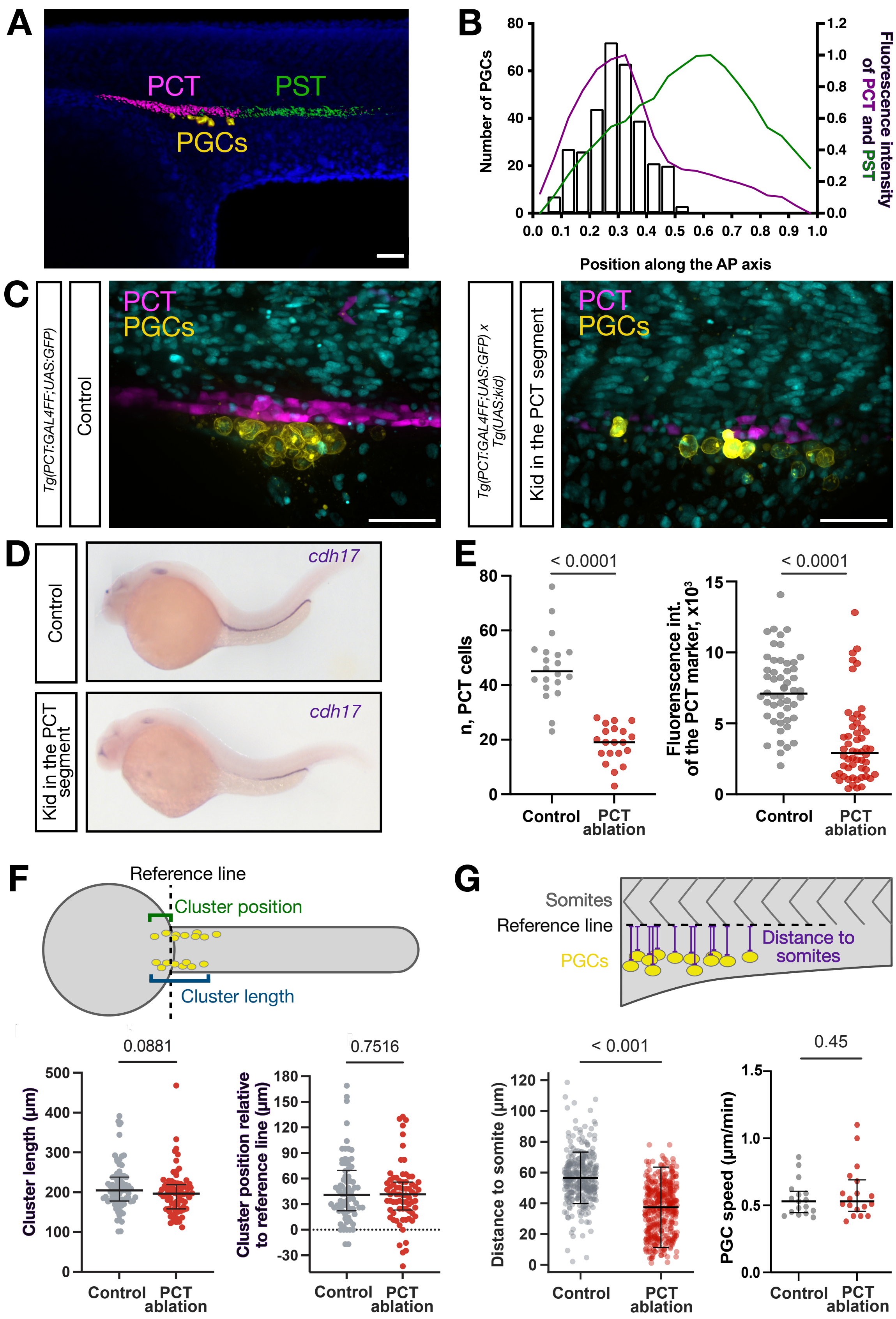

Development • Supplementary information

Fig. S2. PGC clustering upon arrival at the target region aligns with the proximal convoluted tubule region of the developing pronephric duct.

A-B. Quantification of the number of PGCs along the PCT and PST segments. **A.** PGCs were visualized with *Tg(kop:EGFP.F')* and an antisense probe for *egfp* mRNA. The PCT and PST segments were labeled using antisense probes for *nrip2* and *trpm7* mRNAs*,* respectively. Scale bar: 50 µm. **B.** The left y-axis shows the number of PGCs along the two segments. The right y-axis shows the fluorescence intensity of the PCT and PST markers. The x-axis shows the position of PGCs along the PCT and PST segments (as defined by the intensity profiles); here, 0 is the beginning of the PCT segment and 1 is the end of the PST segment. N = 25 embryos and n = 233 PGCs. The analysis was performed at 28 hpf. **C.** A 28 hpf control embryo expressing GFP in the PCT segment (left panel, magenta); a 28 hpf embryo expressing Kid and GFP in the PCT segment indicating significant loss of GFP within the PCT (right panel, magenta). All nuclei are expressing BFP (cyan) and germ cells are expressing farnesylated mCherry (yellow). Scale bar: 50 µm. **D.** mRNA expression pattern of *cdh17* (by WMISH) in control embryos (a representative embryo, left panel) and embryos expressing Kid in their PCT segment at 32 hpf (right panel). 80% of embryos that expressed Kid in the PCT showed a loss of *cdh17* expression in the PCT segment (a representative embryo in the right panel). **E**. Quantification of the PCT ablation by counting the number of the PCT cells (left graph, cells are presented in magenta in the panel **C,** N = 20 embryos for the control and N = 20 embryos analyzed for the PCT ablation) and by measuring mean fluorescent intensity (GFP channel) of the PCT marker (right graph, magenta in the panel **C,** N = 51 embryos for the control and N = 54 embryos analyzed for the PCT ablation). P – Student's t-test, error bars – IQR. Representative images for this experiment are shown in the panel **C**. **F-G.** Illustration of an embryo from the dorsal (**F**) and lateral (**G**) sides depicting the clusters of PGCs (yellow circles). Control embryos (*Tg(PCT:GAL4;UAS:GFP);Tg(kop:lifeact-mCherry)*) and embryos expressing Kid in the PCT (*Tg(PCT:GAL4;UAS:GFP);Tg(kop:lifeact-mCherry);Tg(UAS:kid)*) were assessed for cluster length (**F**, blue line), cluster position (**F**, green line) as well as for the dorso-ventral positioning of the PGCs (**G**, violet lines). For this analysis, we only used embryos that had at least 20% loss of GFP expression within the PCT segment compared to the average GFP intensity within control embryos. **F.** The position of the cluster (green line) was determined by

Development • Supplementary information

the distance from the most anterior PGC to a reference line (dotted line drawn at the cross section of the yolk and the trunk). N = 37 embryos and n = 74 PGC clusters for both control and Kid-expressing embryos. P – Student's t-test, error bars – IQR. **G**. The dorso-ventral positioning of the PGC cluster (left graph) was assessed by measuring the distance (violet lines) to germ cells from the closest ventral somites' border (dotted line). PCT ablation significantly reduced the distance of PGCs from the ventral somite border compared to control (Mann-Whitney U; $p = 6.05e^{-6}$). For statistical analysis, n = 30 PGCs were randomly sampled from each group. Black horizontal lines indicate median distances. Error bars: IQR; N = 52 embryos, n = 341 PGCs for control and N = 62 embryos, n = 397 PGCs for Kid-expressing embryos. Scale bar – 50 µm. Mean PGC speed in control and PCT-ablated embryos (right graph) was calculated from 60 min tracks from 10x time-lapse movies. N = 6 embryos, n = 17 PGCs for control and N = 6 embryos, n = 18 PGCs for the PCT ablation conditions. P – Student's t-test, error bars – IQR. Representative images for this experiment are shown in the panel **C**.

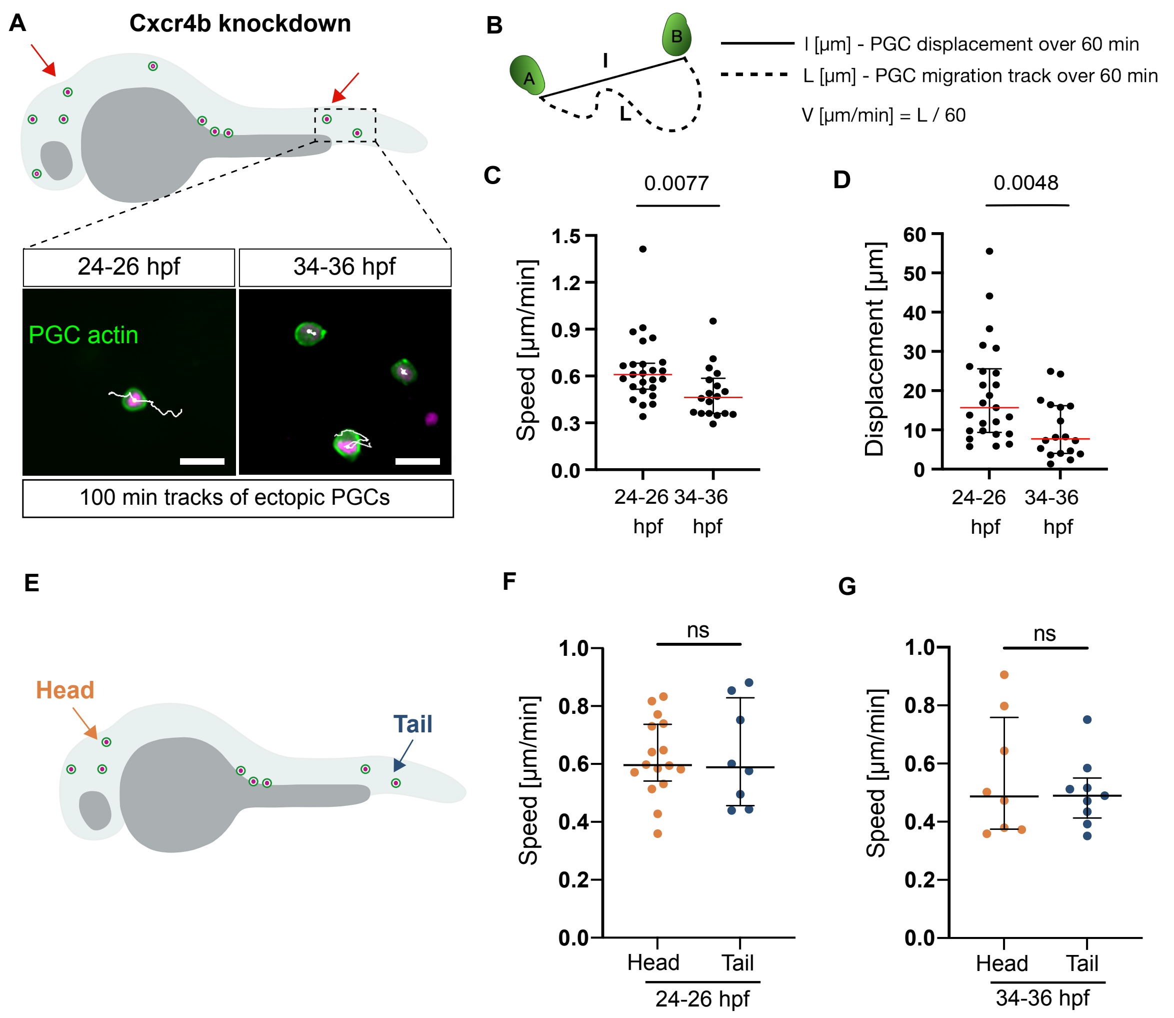

Fig. S3. PGCs' migration speed decay is not due to clustering.

A. Zebrafish embryo schematic with single PGCs located at ectopic regions such as the head and the tail as indicated by the red arrows. The associated images show magnified views of the regions marked by the dotted box, presenting tracks of PGC migration at an ectopic location (e.g., tail region) at 24-26 hpf (left panel) and at 34-36 hpf (right panel). PGCs were labeled by injecting a nuclear RFP-encoding mRNA into *Tg(kop:lifeact-EGFP)* embryos at the one-cell stage. Scale bar – 20 µm. **B.** Scheme illustrating calculation of PGC speed and displacement. Both parameters were calculated from 60 min PGC tracks. Displacement (l) is defined as the minimal distance (in µm) between starting (A) and final (B) PGC positions travelled by the cells during 1 h. Speed (V) is calculated by dividing the entire travel distance (L) over time (60 min). **C.** Migration speed of the ectopic PGCs at 24-26 hpf and 34-36 hpf. **D.** Displacement of the ectopic PGCs at 24-26 hpf and 34-36 hpf. N = 15 embryos, n = 25 PGCs (24-26 hpf); N = 8 embryos, n = 18 PGCs (34-36 hpf). **E.** Zebrafish embryo

Development • Supplementary information

schematic with single PGCs located at ectopic regions such as the head (orange arrow) and the tail (blue arrow). **F-G.** Migration speed of ectopic PGCs located at the head versus migration speed of ectopic PGCs at the tail at 24-26 hpf (**F**) and at 34-36 hpf (**G**). For the head, N = 8, n = 16 PGCs (**F**); N = 6 embryos and n = 9 PGCs (**G**). For the tail, N = 7 embryos and n = 8 PGCs (**F**); N = 4 embryos and n = 9 PGCs (**G**). P – Student’s t-test, error bars – IQR.

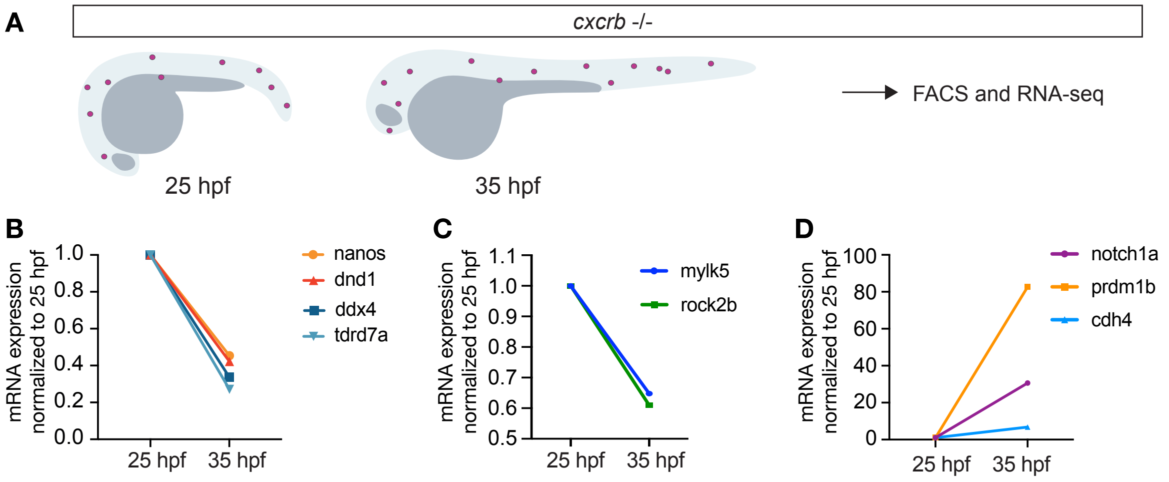

Fig. S4. mRNA levels of proteins important for PGC contractility, specification and differentiation change from 15 to 35 hpf. A. Illustration of 25 and 35 hpf *(cxcr4b*$^{-/-}$*;Tg(kop:mCherry.F’))* embryos used for FACS of PGCs (magenta) and subsequently for obtaining PGC-specific transcriptomes. The normalized mRNA levels of proteins important for specification of germ cells (**B**), for contractility and motility of PGCs (**C**), as well as for the differentiation and adhesion (**D**) are depicted at 25 and 35 hpf.

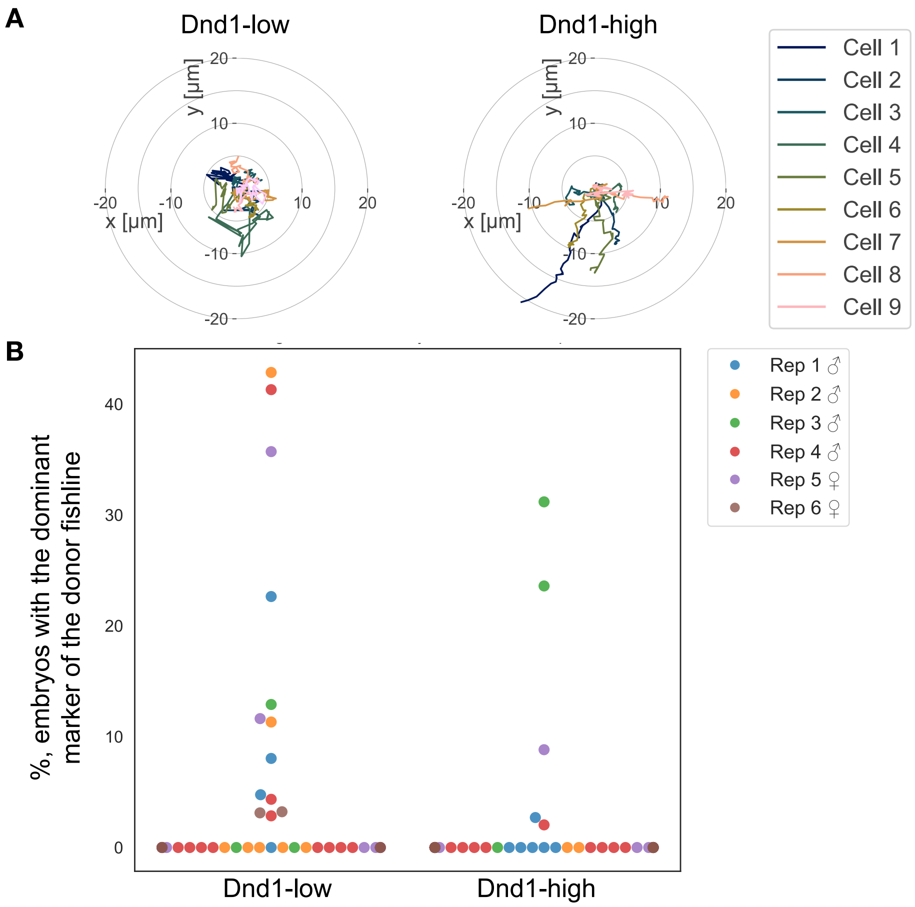

Fig. S5. Reduction of PGC motility at later developmental stages is dependent on the function of Dnd-1 and important for the germline transmission. A. Representative 60-minute tracks of migrating germ cells analyzed in (**Fig. 4B, C**). **B.** Germline transmission efficiency of the transplanted donor germ cells with either normal (Dnd1-low) or high (Dnd1-high) Dnd1 expression levels. Percentage of embryos with the dominant marker of the donor fishline in each offspring clutch from the experiment shown in Fig. 4D. Each dot represents the individual egg clutch of host fish outcross. Colored by replicates (N = 6 replicates; in total 51 males ♂ and 34 females♀ analyzed).

Table S1. RNA-tomography results: Transcripts with expression patterns most similar to *ddx4/vasa*

	Gene	Distance to vasa (24 hpf)	Expression (24 hpf)	Cell Type / Tissue
1	vasa'	0.00	23.21	germ cell
2	'ca15b'	2.18	18.84	germ cell
3	'dnd1'	2.45	21.88	germ cell
4	'nanos3'	2.96	145.98	germ cell
5	'dazl'	3.15	12.32	germ cell
6	'tdrd7a'	3.87	4.40	germ cell
7	'h1m'	4.14	24.46	germ cell
8	'tspan13a'	4.39	55.32	other
9	si:dkey-71l4.5'	4.45	4.01	other
10	'cldn15la'	4.54	13.52	gut
11	'nostrin'	4.55	14.34	whole organism
12	si:rp71-17i16.6'	4.58	19.99	other
13	ogal'	4.99	7.21	other
14	'cfd'	5.07	311.67	pronephric duct, epidermis
15	'TMEM235'	5.11	10.36	other
16	slc4a2a'	5.17	442.71	pronephric duct, PCT
17	slc9a3r1a'	5.20	95.01	pronephric duct
18	slc5a9'	5.20	22.89	pronephric duct, PCT
19	grasp'	5.24	20.89	whole organism
20	zgc:77486'	5.25	13.00	other

Development • Supplementary information

Table S2. Fishlines employed in this work

**Fishline (internal name)**	**Reference**
Wild-type strain of the AB or ABxTL background (WT)	
Tg(kop:EGFP.F'.nos3'UTR-cry: dsRed) (24)	(Blaser et al., 2006)
Tg(kop:lifeact-EGFP.nos3'UTR-cry: dsRed) (38)	(Hartwig et al., 2014)
Cxcr4b ^{t26035} *(ody)*	(Knaut et al., 2003)
Spadetail (Spt ^{b104}*)*	(Ho and Kane, 1990)
Tg(kop:YPet.Ezrin.nos3'UTR.cmlc:EGFP)/ Tg(kop:lifeact-mCherry.nos3'UTR-cry:dsRed) (99/118)	(Olguin-Olguin et al., 2021)
Tg(kop:mCherry.F'.nos3'UTR-cmlc:EGFP) (57)	(Tarbashevich et al., 2015)
Tg(vasa:vasa-EGFP) (81)	(Krøvel and Olsen, 2002)
Tg(buc:buc-EGFP) (180)	(Riemer et al., 2015)
Tg(PCT:GAL4FF;UAS:GFP)/ gSALzGFFD397A (186)	(Kawakami et al., 2010)
Tg(UAS:kid;cmlc:EGFP) (222)	(Labbaf et al., 2022)

Development • Supplementary information

Table S3. DNA constructs employed in this work

Construct	Internal DB number	Reference
mCherry-h2b.globin3'utr	**B325**	(Paksa et al., 2016)
tdTomato-nls.nos3'utr	**C863**	(Olguin-Olguin et al., 2021)
h2a-tagbfp.SV40polyA	**D846**	(Compagnon et al., 2014)
dnd1.globin3'utr	**487**	(Weidinger et al., 2003)
vasaFL.globin3'utr	**333**	(Köprunner et al., 2001)
pCS2-buc	**B639**	(Riemer et al., 2015)
tdrd6.globin3'utr	**F089**	(Hoffmann et al., 2025)
tdrd7.globin3'utr	**A241**	(Hoffmann et al., 2025)
nanos3CDS.globin3'utr	**278**	(Köprunner et al., 2001)
mGFP.nos3'utr	**355**	(Köprunner et al., 2001)

Supplementary References

Blaser, H., Reichman-Fried, M., Castanon, I., Dumstrei, K., Marlow, F. L., Kawakami, K., Solnica-Krezel, L., Heisenberg, C.-P. and Raz, E. (2006). Migration of Zebrafish Primordial Germ Cells: A Role for Myosin Contraction and Cytoplasmic Flow. *Developmental Cell* 11, 613–627.

Compagnon, J., Barone, V., Rajshekar, S., Kottmeier, R., Pranjic-Ferscha, K., Behrndt, M. and Heisenberg, C.-P. (2014). The Notochord Breaks Bilateral Symmetry by Controlling Cell Shapes in the Zebrafish Laterality Organ. *Developmental Cell* 31, 774–783.

Cunningham, F., Achuthan, P., Akanni, W., Allen, J., Amode, M. R., Armean, I. M., Bennett, R., Bhai, J., Billis, K., Boddu, S., et al. (2019). Ensembl 2019. *Nucleic Acids Research* 47, D745–D751.

Dobin, A., Davis, C. A., Schlesinger, F., Drenkow, J., Zaleski, C., Jha, S., Batut, P., Chaisson, M. and Gingeras, T. R. (2013). STAR: ultrafast universal RNA-seq aligner. *Bioinformatics* 29, 15–21.

Hartwig, J., Tarbashevich, K., Seggewiß, J., Stehling, M., Bandemer, J., Grimaldi, C., Paksa, A., Groß-Thebing, T., Meyen, D. and Raz, E. (2014). Temporal control over the initiation of cell motility by a regulator of G-protein signaling. *Proc. Natl. Acad. Sci. U.S.A.* 111, 11389–11394.

Hashimshony, T., Wagner, F., Sher, N. and Yanai, I. (2012). CEL-Seq: Single-Cell RNA-Seq by Multiplexed Linear Amplification. *Cell Reports* 2, 666–673.

Ho, R. K. and Kane, D. A. (1990). Cell-autonomous action of zebrafish spt-1 mutation in specific mesodermal precursors. *Nature* 348, 728–730.

Hoffmann, D., Agranov, T., Kühl, L., Ermlich, L., Reichman-Fried, M., Simons, B. D., Gov, N. S. and Raz, E. (2025). Corrections in single-cell migration path in vivo are controlled by pulses in polar Rac1 activation. *Current Biology* 35, 4365-4382.e8.

Kawakami, K., Abe, G., Asada, T., Asakawa, K., Fukuda, R., Ito, A., Lal, P., Mouri, N., Muto, A., Suster, M. L., et al. (2010). zTrap: zebrafish gene trap and enhancer trap database. *BMC Dev Biol* 10, 105.

Köprunner, M., Thisse, C., Thisse, B. and Raz, E. (2001). A zebrafish *nanos* -related gene is essential for the development of primordial germ cells. *Genes Dev.* 15, 2877–2885.

Krøvel, A. V. and Olsen, L. C. (2002). Expression of a vas::EGFP transgene in primordial germ cells of the zebrafish. *Mechanisms of Development* 116, 141–150.

Labbaf, Z., Petratou, K., Ermlich, L., Backer, W., Tarbashevich, K., Reichman-Fried, M., Luschnig, S., Schulte-Merker, S. and Raz, E. (2022). A robust and tunable system for targeted cell ablation in developing embryos. *Developmental Cell* 57, 2026-2040.e5.

Olguin-Olguin, A., Aalto, A., Maugis, B., Boquet-Pujadas, A., Hoffmann, D., Ermlich, L., Betz, T., Gov, N. S., Reichman-Fried, M. and Raz, E. (2021). Chemokine-biased robust self-organizing polarization of migrating cells in vivo. *Proc. Natl. Acad. Sci. U.S.A.* 118, e2018480118.

Paksa, A., Bandemer, J., Hoeckendorf, B., Razin, N., Tarbashevich, K., Minina, S., Meyen, D., Biundo, A., Leidel, S. A., Peyrieras, N., et al. (2016). Repulsive cues combined with physical barriers and cell–cell adhesion determine progenitor cell positioning during organogenesis. *Nat Commun* 7, 11288.

Patro, R., Duggal, G., Love, M. I., Irizarry, R. A. and Kingsford, C. (2017). Salmon provides fast and bias-aware quantification of transcript expression. *Nat Methods* 14, 417–419.

Riemer, S., Bontems, F., Krishnakumar, P., Gömann, J. and Dosch, R. (2015). A functional Bucky ball-GFP transgene visualizes germ plasm in living zebrafish. *Gene Expression Patterns* 18, 44–52.

Tarbashevich, K., Reichman-Fried, M., Grimaldi, C. and Raz, E. (2015). Chemokine-Dependent pH Elevation at the Cell Front Sustains Polarity in Directionally Migrating Zebrafish Germ Cells. *Current Biology* 25, 1096–1103.

Tarbashevich, K., Ermlich, L., Wegner, J., Pfeiffer, J. and Raz, E. (2023). The mitochondrial protein Sod2 is important for the migration, maintenance, and fitness of germ cells. *Front. Cell Dev. Biol.* 11, 1250643.

Weidinger, G., Stebler, J., Slanchev, K., Dumstrei, K., Wise, C., Lovell-Badge, R., Thisse, C., Thisse, B. and Raz, E. (2003). dead end, a Novel Vertebrate Germ Plasm Component, Is Required for Zebrafish Primordial Germ Cell Migration and Survival. *Current Biology* 13, 1429–1434.

Development • Supplementary information

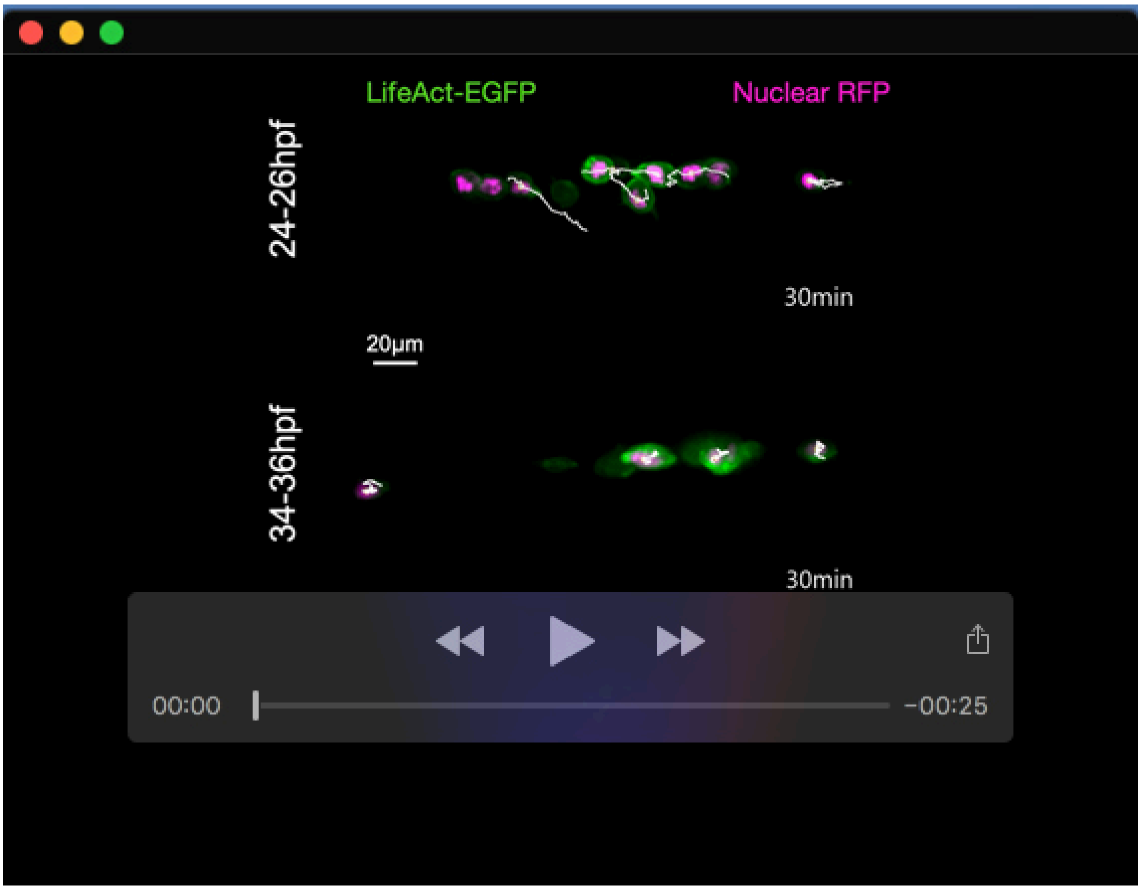

Movie 1. Germ cell migration speed is reduced during development. Time-lapse imaging of PGCs at the gonad region at 24-26 hpf (upper half) and at 34-36 hpf (lower half). PGCs were labeled by injecting nuclear tdTomato-encoding mRNA in *Tg(kop:lifeact-EGFP)* embryos at the one-cell stage.

Development • Supplementary information
